# Relations of Restricted and Repetitive Behaviors to Social Skills in Toddlers with Autism

**DOI:** 10.1007/s10803-021-05014-8

**Published:** 2021-05-06

**Authors:** Pang Chaxiong, Catherine Burrows, Kelly N. Botteron, Stephen R. Dager, Annette M. Estes, Heather C. Hazlett, Robert T. Schultz, Lonnie Zwaigenbaum, Joseph Piven, Jason Wolff, IBIS Network

**Affiliations:** 1 University of Minnesota, 56 East River Road, Minneapolis, MN 55455, USA; 2 Washington University in St. Louis, 660 S Euclid Ave, St. Louis, MO 63110, USA; 3 University of Washington, 1701 NE Columbia Rd, Seattle, WA 98195, USA; 4 University of North Carolina at Chapel Hill, 321 S Columbia St, Chapel Hill, NC 27516, USA; 5 Children’s Hospital of Philadelphia, 3401 Civic Center Blvd, Philadelphia, PA 19104, USA; 6 University of Alberta, 116 St & 85 Ave, Edmonton, AB T6G 2R3, Canada

**Keywords:** Autism, Restricted repetitive behavior, Insistence on sameness, Repetitive sensory-motor, Self-injurious behavior, Social skills

## Abstract

We examined the relations of restricted and repetitive behaviors (RRB; insistence on sameness, repetitive sensory-motor, self-injurious behavior) to social skills overall and aspects that comprise social skills as measured by the VABS-II (coping skills, play/leisure time, interpersonal relationships) in 24- (*n* = 63) and 36-month old (*n* = 35), high-familial-risk toddlers with ASD. Hierarchical linear regression results indicated that repetitive sensory-motor was the best predictor of social skills overall. Secondary results indicated that all three RRB subtypes were associated with each subdomain of social skills; however, repetitive sensory-motor was the strongest and most consistent among these effects. While our results suggests a general negative relation of subtypes of RRB to aspects of adaptive social function, repetitive sensory-motor behaviors may be of particular relevance to the development of social skills during toddlerhood.

Although restricted repetitive behaviors (RRB) and social deficits are both diagnostic domains of autism spectrum disorder (ASD), previous research has predominantly focused on social deficits (Richler et al., 2010; Troyb et al., 2016). Much less is known of the developmental trajectories and outcomes related to RRB and even less so concerning the interplay of RRB and social deficits (Harrop et al., 2014; Richler et al., 2010). As both are core features of ASD and co-emerge in the first several years of life, there is a need to characterize their influence on one another in early childhood (Leekam et al., 2011). Previous work has found that RRB are negatively associated with social communication skills in early childhood (e.g., Bruyneel et al., 2019; Larkin et al., 2017; Mirenda et al., 2010; Watt et al., 2008; Wolff et al., 2014); however, empirical evidence of this link continues to be limited and mixed (e.g. Harrop et al., 2014; Thorup et al., 2018). Moreover, much of this rather limited body of work has considered both RRB and social ability as unitary constructs without regard to relations among subtypes or subdomains. Examining whether specific forms of RRB are differentially associated with aspects of early social functioning would further elucidate this relation by informing whether RRB and social deficits are characterized by specific or general phenomena. This knowledge is important for understanding how RRB and social deficits co-emerge and change over time, perhaps informing the developmental process culminating in a diagnosis of ASD. Thus, the purpose of this study was to examine the relations between specific subtypes of RRB and social skills in 24- and 36-month-olds with ASD.

Restricted and repetitive behaviors refer to stereotyped or repetitive motor movements, use of objects, or speech; insistence on sameness, inflexible adherence to routines, or ritualized behavior; and highly fixated interests (American Psychiatric Association [APA], 2000). More recently, unusual sensory interests or hyper- or hypoactivity to sensory input were introduced to this domain (APA, 2013). Despite the heterogeneity of RRB, previous studies indicate that topographies of RRB load onto superordinate categories. The most consistent of these in the literature are factors described as insistence on sameness (IS) and repetitive-sensory motor (RSM) (Bishop et al., 2013; Hiruma et al., 2021; Uljarević et al., 2017). IS refers to compulsive, ritualistic behaviors and resistance to changes in routines, whereas RSM refers to apparently purposeless, stereotyped movements and preoccupations with a specific interest, activity, or object. Validation studies of the Repetitive Behavior Scale – Revised (RBS-R; Bodfish et al., 2000) specifically have identified an additional factor, self-injurious behavior (SIB), which stems from a subscale unique to that measure (Bishop et al., 2013; Lam & Aman, 2007; Mirenda et al., 2010). SIB refers to repetitive behaviors that cause or have the potential to cause bodily injury ranging from minor to severe. Thus, a three factor model of RRB derived from the RBS-R (i.e., IS, RSM, and SIB) has been recommended for studies of young children with ASD (Mirenda et al., 2010).

Despite being a core feature of ASD, RRB are not unique to individuals with ASD and are also present in individuals with other disabilities as well as those who are typically-developing (Leekam et al., 2011). The accumulation of RRB studies reveal, however, that individuals with ASD present with a higher frequency and severity of RRB and a greater variety of topographies by age two and three (e.g., Cox et al., 1999; Guthrie et al., 2013; Kim & Lord, 2010; Rogers et al., 2003; Wetherby et al., 2004) and potentially as early as 6–12 months (Baranek, 1999; Elison et al., 2014; Purpura et al., 2017; Wolff et al., 2014; Zwaigenbaum, 2005). And, whereas RRB in typically-developing toddlers are assumed to scaffold the learning of more complex skills such as volitional motor activity, their persistence in toddlers with ASD may have the inverse effect by interfering with early opportunities to advance cognitive and social development (Charman et al., 2005; Harrop et al., 2014; Stronach & Wetherby, 2014; Troyb et al., 2016; Wolff et al., 2014). Specifically, elevated engagement in RRB in the early years of life may compete with and limit opportunities to engage in social interaction, contributing to the accumulation of social skill deficits.

With regard to social skills, prospective studies of infants siblings of children with ASD, who are at elevated familial-risk for ASD, suggest that defining social features of ASD may not become evident until the latter half of the second year of life (Ozonoff et al., 2010), though more subtle social differences have been reported (e.g. Sacrey et al., 2018). In a recent longitudinal study of adaptive behavior among infant siblings, Sacrey et al. (2019) found that social skills as measured by the Vineland Adaptive Behavior Scale-II (VABS-II; Sparrow et al.., 2005) were in the average range for all siblings regardless of later diagnostic outcome, with trajectories gradually diverging over toddlerhood. Considered alongside prospective studies of RRB in infant siblings (e.g. Elison et al., 2014; Wolff et al., 2014), such findings suggest that differences in RRB may precede significant deviations in social skills and raise the possibility of reciprocal developmental effects.

However, the relation of RRB to social skills in early childhood is not well understood and empirical data are limited. Studies using direct observations of child behaviors (e.g., Communication and Symbolic Behavior Scales Developmental Profile [CSBS]) have found that repetitive and stereotyped behaviors were negatively associated with social competence and social affect among toddlers with ASD (Morgan et al., 2008; Stronach & Wetherby, 2014; Watt et al., 2008). Ozonoff et al. (2008) found that restricted and repetitive play behaviors (e.g., spinning and unusual visual exploration of objects) were positively associated with communication and social impairments for 12-month-olds who were later diagnosed with ASD. Relatedly, Bruckner and Yoder (2007) found that restricted use of objects in toddlers with ASD was negatively associated with response to joint attention and coordinated attention to an object and person. Using parent-report measures, Schertz et al. (2016) examined the relation between RRB (RBS-R) and social skills (VABS-II) in a sample of 2-years olds with ASD. They found negative correlations between all RRB subtypes and socialization domain scores. Using the same measures with a prospective, high-risk sample of toddlers with ASD, Wolff et al. (2014) found a similar relation between RRB and socialization domain scores at 24 months and further found that RRB was not related to other domains of adaptive behavior. These findings suggest a rather specific behavioral association that may reflect developmental effects unique to early childhood. Moreover, the inverse relation of RRB to social skills may not extend to children who are typically developing. While IS and RSM behaviors may be common among such children during early childhood, there is evidence that aspects of social skills, such as frequency and sophistication of play behavior, are not associated with RRB (Honey et al., 2007).

It is still unclear, however, if the RRB-social skills relation is driven by specific aspects of one or both domains. Finding that a particular subtype of RRB and/or aspect of social skills is related above and beyond others would point to specific phenomena for further study. For example, it may be that the general relation reported in the literature is largely driven by an inverse correlation between specific aspects of RRB and social function (e.g. repetitive motor behavior and play sophistication). In contrast, the absence of significant differentiated relations would suggest a general phenomenon, such that multiple aspects or topographies of RRB are inversely related to an array of social skills in toddlerhood. This knowledge is important for informing our understanding of how RRB and social deficits co-develop during the early unfolding of ASD and may inform specific targets for early intervention.

In the present study, we sought to further elucidate the relation between RRB and social skills in young children with ASD previously reported in Wolff et al. (2014) by examining whether this relation is characterized by specific or general phenomena. To accomplish this, we examined whether subtypes of RRB (IS, RSM, SIB) were differentially associated with (1) social skills overall (VABS-II socialization domain score) and (2) specific aspects that comprise the socialization domain in children with ASD at ages 24 and 36 months. The subdomains of behavior comprising the construct of socialization as defined by the VABS-II include coping skills (CS), play and leisure (PL), and interpersonal relationships (IPR). We hypothesized that higher RRB subtype scores (higher RRB severity) would be associated with lower socialization domain scores at both 24 and 36 months. We further hypothesized that RSM would drive this relation (i.e., explain significant variation in socialization domain scores at both time points). These hypotheses were based on findings from Wolff et al. (2014) as well as studies of older children (e.g. Lampi et al., 2018) which suggest that components of RSM are inversely associated with social skills. We did not have hypotheses about relations between RRB subtypes and specific aspects of social skills as this was exploratory.

## Methods

### Participants

Participants were from the ongoing Autism Center of Excellence (ACE) Network Infant Brain Imaging Study (IBIS). IBIS participants were recruited through research registries, websites, email blasts, flyers, brochures, and community clinics. Exclusion criteria included having (a) genetic or medical conditions that impacted development; (b) significant visual or hearing impairments; (c) a birth weight < 2000 g and/or a gestational age < 36 weeks or significant perinatal adverse experiences; (d) contraindication for MRI; (e) a home language other than English; (f) first-degree relatives with psychosis, schizophrenia, or bipolar disorder; and being (g) a twin, adopted or a half-sibling of a proband. For eligible participants, longitudinal data on cognitive development, adaptive functioning, and ASD characteristics were collected at four time points (i.e., 6, 12, 24, 36 months of age). At each time point, participants were given two $50 incentives for participation in a behavioral assessment and magnetic resonance imaging, in addition to travel reimbursements. Recruitment, screening, and assessment of IBIS participants were conducted at four clinical sites: University of North Carolina, Children’s Hospital of Philadelphia, University of Washington, and Washington University in St. Louis. Each clinical site received study approval from their Human Subjects Review Board. Written informed consent was obtained and documented for all participants.

Of the IBIS dataset, our study included high-familial-risk participants with completed RBS-R, VABS-II, and Mullen Scales of Early Learning (MSEL) assessments at 24 and/or 36-months who met clinical best-estimate diagnostic criteria for ASD based on DSM-IV-TR criteria. Given the recurrence rate of ASD in families, following younger siblings of children with ASD allows for the prospective study of infancy and toddlerhood in children who are later diagnosed. High-risk toddlers were defined as such by virtue of having an older sibling with an existing diagnosis of ASD. Proband diagnoses were confirmed using the Social Communication Questionnaire and Autism Diagnostic Interview-Revised (ADI-R; Lord et al. 1994). For their younger siblings (current participants), diagnoses were based on multiple assessments (i.e., Autism Diagnostic Observation Scale, ADI-R, and MSEL) conducted at 24 and/or 36 months of age that were independently verified by a senior clinician blind to classification. This resulted in a final study sample of sixty-three 24- and thirty-five 36-month-olds with ASD. Twenty-six (74%) participants from the 36-month sample were part of the 24-month sample. Participant descriptive information are presented in Table 1.

### Measures

#### Repetitive Behavior Scale-Revised (RBS-R; Bodfish et al., 2000)

The RBS-R is a parent rating scale that measures RRB based on parent/caregiver observations over the last month that has been used in samples of individuals with ASD from early childhood to adulthood (e.g., Antezana et al., 2019; Boyd et al., 2010; Gabriels et al., 2008; McDermott et al., 2020; Oakes et al., 2016; Schertz et al., 2016; Wolff et al., 2014, Wolff et al., 2019). It consists of 43 items that correspond to six RRB subscales: stereotyped, self-injurious, compulsive, ritualistic, sameness, and restricted. Subscale scores are calculated by summing the ratings (0 = “behavior does not occur” to 3 = “behavior is a severe problem”) of all their correspondent items, representing the severity of each type of RRB. An overall RRB score is calculated by summing the subscale scores. The RBS-R has been shown to have good to excellent psychometric properties, including evidence of reliability and construct validity, particularly for derived three- and five-factor models (Scahill et al., 2015). In the present sample, there was strong internal consistency (0.80 to 0.91) for items comprising RSM and IS factors at both 24 and 36 months. Internal consistency for SIB, however, was poor (0.50 to 0.55), which may reflect the relatively fewer items or lower endorsement/lower variability for this factor. This aspect of construct validity should be considered in the interpretation of SIB-related results within this sample.

To feasibly examine distinct relations between RRB and social skills, we elected to examine RRB items in three subtypes, following the three-factor model recommended by Mirenda et al. (2010) for fit and parsimony: insistence on sameness (IS), repetitive sensory-motor (RSM), and self-injurious behavior (SIB). Items from the compulsive, ritualistic, and sameness subscales were combined to create the IS subtype, and items from the stereotyped and restricted behavior subscales were combined to create the RSM subtype. The SIB subtype consisted of items from the self-injurious subscale. IS, RSM, and SIB subtype scores were calculated by summing their correspondent subscale scores.

There were several reasons for adopting a three-factor model to individual RRB items. First, examining individual items exacerbates the multiple comparisons problem, while also posing a multicollinearity issue. Additionally, collapsing similar RRB items into larger categories increases reliability and also eases cross-study communication of RRB and may be more useful for diagnostic and intervention purposes. Thus, we determined that a three-factor model would allow for feasible examination of distinct relations between RRB and social skills without inflating type one error rates, while acknowledging that alternative RRB factor structures are plausible.

#### Vineland Adaptive Behavior Scales-II (VABS-II; Sparrow et al., 2005)

The VABS-II is a standardized, norm-referenced, semi-structured parent/caregiver interview that assesses daily adaptive behavior in individuals from birth to 90 years of age. Our analyses utilize the socialization domain score (*M* = 100, *SD* 15, range = 0–160) and the CS, PL, and IPR subdomain scores (*M* = 15, *SD* 3, range = 0–25). The VABS-II provides both standard and age-equivalent scores; however, only standard scores were used in this study. Strong reliability and validity of the VABS-II have been demonstrated, including internal consistency ranging from 0.84 to 0.92 for the Socialization domain and good to excellent test–retest reliability (Sparrow et al., 2005). Previous studies of young children with ASD as well as high-risk infant sibling samples suggest good sensitivity (Chatham et al., 2018) and predictive (Sacrey et al., 2019) and convergent validity (Dupuis et al., 2020) for socialization domain and subdomain scores.

#### Mullen Scales of Early Learning (MSEL; Mullen, 1995)

We used the Mullen Early Learning Composite (Mullen ELC) score from the MSEL as a proxy of general cognitive ability. The MSEL is a standardized developmental assessment consisting of five brief scales that describe early cognitive and motor development in children birth to 68 months of age. The MSEL was administered by trained research staff; reliability across all study sites was established prior to administration and was maintained throughout data collection.

### Statistical Analyses

#### Research Question 1

**Fig. 1:**
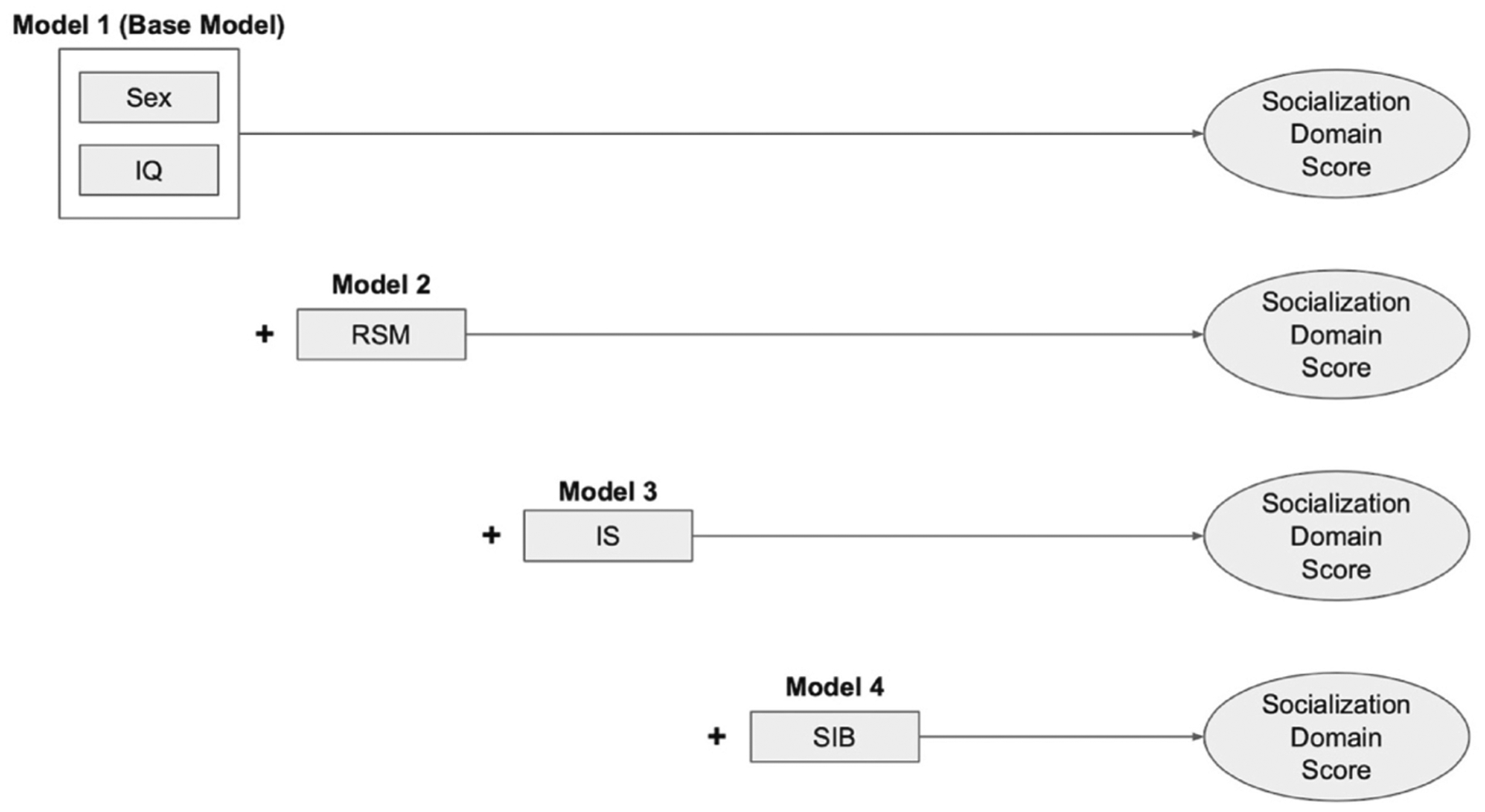
Illustration of 4-step hierarchical linear regression *Note*. IS = insistence on sameness; RSM = repetitive sensory motor; SIB = self-injurious behavior; IQ = Mullen Early Learning Composite.

Simple correlations were generated to describe binary relations between each RRB subtype (IS, RSM, SIB) and socialization domain score. We then conducted a four-step hierarchical linear regression to examine whether different RRB subtypes explained unique variance in socialization domain scores at 24 and 36 months. Mixed effects regression was considered given the potential for age effects; however, the addition of an age interaction term was not significant for any model, and thus was not used for the analyses presented herein. Sex and Mullen ELC score were entered at Step 1 as covariates, serving as our base model. Sex was included given previous evidence of differential effects with type or frequency of RRB (Kim & Lord, 2010; Knutsen et al., 2019). Mullen ELC score was included given evidence that general cognitive ability may be negatively or positively associated with different RRB subtypes (Militerni et al., 2002; Richler et al., 2010). The moderating effect of Mullen ELC score was further evaluated as an interaction term. However, this did not improve fit for any model, and thus was entered as a main effect only. RSM was entered at step 2 (Model 2), followed by IS at step 3 (Model 3) and SIB at step 4 (Model 4; see Fig. 1). This order was determined in alignment with our hypothesis that RSM would drive the RRB-social skills relation. Change in R^2^ was examined to determine whether models with the addition of each RRB subtype explained additional variation in socialization domain scores above and beyond the previous model.

#### Research Question 2

Next, we conducted multiple linear regressions to explore whether RRB subtypes were differentially associated with socialization subdomains (CS, PL, IPR) at 24- and 36-months. Mullen ELC was included as a covariate in multiple linear regressions for 36-month-old data, as it was a significant predictor of socialization domain scores at this age in all models from Research Question 1.

Standardized RRB subtype scores were used in all analyses. To account for multiple comparisons in research questions one and two, results are presented with Bonferroni adjusted alpha levels (*α* = 0.008 and *α* = 0.003, respectively) and without (*α* = 0.05). This analysis plan was determined after careful examination of assumptions for conducting linear regressions. Density and scatter plots of residuals indicated that assumptions of normality, linearity, and homoscedasticity were tenable. All analyses were conducted in the R software (R Core Team, 2019).

## Results

### Research Question 1

We first asked whether RRB subtypes (IS, RSM, SIB) were differentially associated with socialization domain scores (social skills overall) among high-risk children with ASD at 24 and 36 months. It was hypothesized that higher RRB subtype scores would be associated with lower socialization domain scores at both 24 and 36 months, with RSM explaining significant variation in socialization domain scores at both time points. As expected, correlations between RRB subtypes and socialization domain scores were negative and relatively consistent at both 24 months, IS (r = − 0.51, 95% CI [− 0.29, − 0.67]); RSM (r = − 0.53, 95% CI [− 0.32, − 0.69]); SIB (r = − 0.39, 95% CI [− 0.16, − 0.58]); and 36 months, IS (r = − 0.46, 95% CI [− 0.15, − 0.69]); RSM (r = − 0.66, 95% CI [− 0.42, − 0.81]); SIB (r = − 0.39, 95% CI [− 0.07, − 0.64]). Hierarchical linear regression results presented in Table 2 reveal negative relations between severity of RRB subtypes and socialization domain scores at both ages. The addition of RSM to our base model (sex and Mullen ELC) best explained variation in socialization domain scores; subsequent models with IS and SIB did not explain variation above and beyond the model with RSM alone.

#### 24 Months

For 24-month-olds, the base model with sex and Mullen ELC score accounted for *R*^2^ = 0.03 and, as a full model, did not differ significantly from zero, F(2, 60) = 0.8, *p* = 0.438. Neither sex (β = − 1.15, *p* = 0.699) nor Mullen ELC (β = 1.47, *p* = 0.239) were significant predictors of socialization domain scores. In Model 2, RSM was added as a predictor, accounting for *R*^2^ = 0.30 and, as a full model, did differ significantly from zero, F(3, 59) = 8.3, *p* < 0.001. The addition of RSM resulted in a significant change in *R*^2^, Δ*R*^2^ = 0.27, F(1, 59) = 22.7, *p* < 0.001, which remained significant at the adjusted alpha level (*α* = 0.008). Controlling for sex and Mullen ELC, RSM was a significant predictor of socialization domain scores (β = − 5.08, *p* < 0.001). In Model 3, IS was added as a second predictor, accounting for *R*^2^ = 0.33 and, as a full model, did differ significantly from zero, F(4, 58) = 7.2, *p* < 0.001. The addition of IS did not result in a significant change in *R*^2^, however, Δ*R*^2^ = 0.04, F(1, 58) = 3.08, *p* = 0.085. Controlling for sex, Mullen ELC and RSM, IS was not a significant predictor of socialization domain scores (β = − 3.21, *p* = 0.085). In Model 4, SIB was added as a third predictor, accounting for *R*^2^ = 0.34 and, as a full model, did differ significantly from zero, F(5, 57) = 5.7, *p* < 0.001. The addition of SIB also did not result in a significant change in *R*^2^, Δ*R*^2^ < 0.01, F(1, 57) = 0.15, *p* = 0.696. Controlling for sex, Mullen ELC, RSM and IS, SIB was not a significant predictor of socialization domain scores (β = − 0.61, *p* = 0.696).

#### 36 Months

For 36-month-olds, the base model with sex and Mullen ELC score accounted for *R*^2^ = 0.32 and, as a full model, did differ significantly from zero, F(2, 32) = 7.7, *p* = 0.002. Sex was not a significant predictor of socialization domain scores (β = 4.34, *p* = 0.326); however, Mullen ELC was (β = 0.34, *p* < 0.001). In Model 2, RSM was added as a predictor, accounting for *R*^2^ = 0.60 and, as a full model, did differ significantly from zero, F(3, 31) = 8.3, *p* < 0.001. The addition of RSM resulted in a significant change in *R*^2^, Δ*R*^2^ = 0.27, F(1, 31) = 20.7, *p* < 0.001, which remained significant at the adjusted alpha level (α = 0.008). Controlling for sex and Mullen ELC, RSM was a significant predictor of socialization domain scores (β = − 6.31, *p* < 0.001). In Model 3, IS was added as a second predictor, accounting for *R*^2^ = 0.60 and, as a full model, did differ significantly from zero, F(4, 30) = 11.1, *p* < 0.001. The addition of IS did not result in a significant change in *R*^2^, however, Δ*R*^2^ < 0.01, F(1, 30) = 0.1, *p* = 0.821. Controlling for sex, Mullen ELC and RSM, IS was not a significant predictor of socialization domain scores (β = 0.50, *p* = 0.821). In Model 4, SIB was added as a third predictor, accounting for *R*^2^ = 0.62 and, as a full model, did differ significantly from zero, F(5, 29) = 9.4, *p* < 0.001. The addition of SIB also did not result in a significant change in *R*^2^, Δ*R*^2^ = 0.02, F(1, 29) = 1.8, *p* = 0.193. Controlling for sex, Mullen ELC, RSM and IS, SIB was not a significant predictor of socialization domain scores (β = − 2.14, *p* = 0.193).

Altogether, the results reveal that higher RRB severity was associated with lower parent-reported social skills. Furthermore, Model 2 with RSM best predicted social skills overall. Models 3 and 4 with IS and SIB, respectively, did not predict social skills overall above and beyond that of Model 2. This was the case at both 24 and 36 months of age. As covariates, general cognitive ability played a more significant role at 36 than at 24 months while sex did not appear to play a meaningful role at either time points.

### Research Question 2

We next explored the relations between each RRB subtype and the three subdomains of socialization (CS, PL, IPR). Mullen ELC score was included as a covariate in multiple linear regressions at 36 months based on its significance as a predictor in socialization domain scores from research question one. Unadjusted regression results in Table 3 suggest that the three RRB subtypes were related to the three socialization subdomains across 24 and 36 months; adjusted regression results, however, suggest that RSM best predicted socialization subdomains at both time points.

#### 24 Months

For 24-month-olds, unadjusted regression results indicated that IS significantly predicted CS (β = − 0.71, *p* = 0.006), PL (β = − 0.90, *p* = 0.004), and IPR (β = − 0.90, *p* < 0.001); however, IS was a significant predictor of only IPR at the adjusted alpha level (*α* = 0.003). RSM significantly predicted CS (β = − 0.59, *p* = 0.024), PL (β = − 1.29, *p* < 0.001) and IPR (β = − 0.76, *p* = 0.002). At the adjusted alpha level, RSM remained a significant predictor of PL and IPR. SIB significantly predicted PL (β = − 0.76, *p* = 0.015) and IPR (β = − 0.75, *p* = 0.003) but did not significantly predict CS (β = − 0.40, *p* = 0.163). At the adjusted alpha level, SIB remained a significant predictor of only IPR.

#### 36 Months

For 36-month-olds, regression results indicated that IS significantly predicted CS (β = − 0.68, *p* = 0.030), PL (β = − 0.93, *p* = 0.031) and IPR (β = − 0.80, *p* = 0.017), controlling for Mullen ELC. At the adjusted alpha level (*α* = 0.003), however, IS was no longer a significant predictor of any socialization subdomain. RSM also significantly predicted CS (β = − 1.06, *p* < 0.001), PL (β = − 1.51, *p* < 0.001) and IPR (β = − 0.77, *p* = 0.028), controlling for Mullen ELC. At the adjusted alpha level, RSM remained a significant predictor of CS and PL. SIB significantly predicted CS (β = − 0.67, *p* = 0.035) and PL (β = − 0.90, *p* = 0.040) but did not significantly predict IPR (β = − 0.31, *p* = 0.375), controlling for Mullen ELC. At the adjusted alpha level, SIB was no longer a significant predictor of any socialization subdomain.

Overall, unadjusted results reveal that the three RRB subtypes were significant predictors of the three aspects of social skills, with SIB being a less consistent predictor across the two time points. Adjusted results indicate that RSM was the only RRB subtype to significantly predict two of three aspects of social skills at both 24 and 36 months.

#### Follow-Up Analyses

To ensure that our adoption of a three-factor RRB model did not mask distinct relations between RRB and socialization subdomains, we conducted a follow-up series of analyses using as predictors the six subscale breakdown from the RBS-R (i.e., stereotyped, self-injurious, compulsive, ritualistic, sameness, and restricted). Analyses with the six RBS-R subscales yielded results that were consistent with the three-factor model used in our primary analysis. Standardized regression analyses are provided in supplemental Tables 1 and 2.

## Discussion

Motivated by previous findings (Wolff et al., 2014), we sought to determine whether the negative relation between RRB and social skills is characterized by specific or general phenomena by examining whether subtypes of RRB are differentially associated with social skills overall and aspects of the domain in toddlers with ASD. Our results suggest that RSM may drive the RRB-social skills relation. First, our base model with the addition of RSM explained a significant amount of variation in social skills overall, while the subsequent addition of IS and SIB did not contribute significantly. Secondly, although each of the three RRB subtypes were negatively associated with at least one social skill subdomain at 24 and 36 months, RSM was characterized by the greatest effect sizes and most consistently survived adjustment for multiple comparisons. This was particularly true for the relation of RSM to play and leisure skills, which is perhaps unsurprising given the rapid development of play over this age range. In summary, while we identified that while RSM, IS, and SIB were negatively associated with at least some aspects of social skills in familial-risk toddlers who developed ASD, RSM appears to most consistently explain variance in adaptive social behavior.

The present findings are consistent with reports showing that RSM is most prominent during toddlerhood but begins to gradually decline by preschool age in children with ASD (Lord et al., 2015; Richler et al., 2007). Conversely, these same studies indicate that IS remains relatively steady or gradually increases into school age. Given that patterns of RRB change across development, we expect that so to do their relation adaptive social function. With collection of school-age follow-up currently underway, we will soon be able to examine whether and how these relations change with age. Interestingly, the contribution of cognitive ability in our modeling was trivial at age 24 months but increased in effect by age 36 months. This is consistent with our previous findings (Wolff et al., 2014) as well as work by others suggesting that the role of cognitive ability to both social skills and RRB development may strengthen from late toddlerhood onward (Bishop et al., 2006; Tomaszewski et al., 2020).

As RRB and social communication deficits are both core features of ASD, understanding whether and how one impacts the development of the other could inform the early unfolding of ASD. Although a causal direction is unclear, one possibility is that children with ASD present with social communication deficits and, thus, engage in RSM and other forms of RRB to occupy time not filled by competing social interactions or typical play behavior (Lampi et al., 2018; Ray-Subramanian & Ellis Weismer, 2012). Relatedly, and in consideration of the young age of our participants, it is possible that early RRB, irrespective of topography, interferes with the acquisition of social skills. Emergent literature suggests that RRB may be more than independent, passive symptoms of ASD and instead potentially contribute to its emergence by encroaching on opportunities to develop and practice adaptive social behaviors, thereby contributing to social deficits (Richler et al., 2010). This possibility is now more viable with accumulating research indicating that timing of RRB emergence precedes marked social deficits. Elison et al. (2014), for example, found that RRB are observed in 1-year old children who are later diagnosed with ASD, prior to reported divergence in social skills (Ozonoff et al., 2010). Notably, these RRB occurred in the context of standardized social communicative presses, often in place of an expected response. Furthermore, it is possible that the relation between RRB and social skills begins as unidirectional, becoming bidirectional– and mutually sustaining– over development (i.e., RRB perpetuate social deficits, and social deficits in turn precipitate RRB). An example of this might be repetitive behaviors that take on the function of a coping mechanism in lieu of more conventional responses (Manor-Binyamini & Schreiber-Divon, 2019), a possibility supported by our finding that RRB and coping skills are negatively associated, with the effect strengthening from 24 to 36 months.

Finally, despite strong negative associations, it is also possible that RRB and social skill deficits co-occur but do not contribute to the development of one another. By definition, both features are present in individuals with ASD; this alone does not imply an interaction or causal relation. The present work cannot rule out the possibility that these aspects of ASD develop independently. However, experimental studies demonstrating that engaging in social behavior resulted in reduced rates of RRB suggest that RRB and social interactions may be incompatible, wherein engagement in one set of behaviors reduces opportunity to engage in the other. For example, Lee et al. (2007) found that play initiation from typically-developing peers led to an increase in the percentage of time elementary children with ASD were socially engaged and a simultaneous decrease in their stereotypic behaviors. Lampi et al. (2018) found that school-aged children with ASD displayed the fewest number of RRB during experimental tasks in which they were highly engaged in social and motor behaviors. Similar observations of an increase in social behavior and a simultaneous decrease in RRB were also found in earlier studies examining school-aged children with ASD (e.g., Donnellan et al., 1984; Lee & Odom, 1996; Lord & Hopkins, 1986). Such a collateral relation offers some evidence that RRB and adaptive social function likely influence one another.

### Limitations

This study has several limitations. Our sample size at 36 months (*n* = 35) is half the size of our sample at 24 months (*n* = 63), and thus provided less power to detect a small or even medium effect, potentially resulting in a type II error. Relatedly, the majority of participants in both samples were male thus limiting conclusions that can be drawn about the effects of sex. Follow-up work with larger samples, including girls with ASD, are warranted. Only 13 of 60 socialization items on the VABS-II are appropriate for children under 36 months and are unevenly distributed: seven pertaining to IPR, four pertaining to PL, and two pertaining to CS. While there was sufficient variability in subdomain scores, the low number of items contributing to each subscale, particularly for CS, presents validity concerns that should be considered when interpreting the results regarding specific relations between RRB and socialization subdomains. It is clear that the results of the exploratory analysis of socialization subdomains requires follow-up with additional measures of social behavior suitable for this age group. Relatedly, the RBS-R is not specific to children in this age range and, thus, parents were asked to respond to items that may not have been developmentally appropriate for their child. Future studies should consider quantitative measures of RRB that are specifically designed for very young children. Furthermore, we examined the relation between RRB and social skills at a sub-categorical level (i.e., three RRB subtypes and three socialization subdomains) as opposed to item level. It is possible that a three-factor RRB model and the VABS-II breakdown of social skills masks important item-level relations. Future studies might consider examining theory-driven item-level relations using structural equation modeling to build upon the present findings.

## Conclusions

Although differences in social function and RRB represent diagnostic characteristics of ASD, both have largely been studied separately, and their relation to one another is not well understood. Previous studies have found negative associations between RRB and social skills; however, whether this relation is characterized by general or specific phenomena has been unclear. Our study provides additional evidence that RRB and adaptive social skills may develop in inverse relation to one another at ages two and three. Indeed, it would appear that each RRB subtype is associated with at least some aspect of social skills. Nonetheless, RSM appears to best explain variance in social skills during toddlerhood and may represent a specific target for further study. Striving to characterize the causal direction and nuances of this relation may enable more effective and targeted intervention for supporting adaptive social function by elucidating the role of RRB as well as contribute to our understanding of the early emergence of ASD.

## Supplementary Material

Supplementary Tables

## Figures and Tables

**Table 1:** Participant characteristics

Characteristic	24-Months-olds	36-Month-olds
*N*	63	35
Age	24.69 (1.47) [23.40–32.10]	39.03 (4.59) [34.80–57.50]
Sex		
Male	49 (77.8%)	29 (82.9%)
Female	14 (22.2%)	6 (17.1%)
RRB (RBS-R)		
IS	5.16 (8.74) [0–61]	10.23 (8.73) [1–38]
RSM	3.73 (4.31) [0–20]	6.29 (5.12) [0–18]
SIB	1.27 (1.92) [0–10]	1.34 (2.55) [0–13]
Total	10.16 (13.93) [0–91]	17.86 (14.40) [2–55]
Socialization (VABS-II)		
CS	14.00 (2.08) [8–19]	11.77 (8.73) [8–15]
PL	13.00 (2.51) [8–19]	11.97 (2.72) [8–16]
IPR	12.48 (2.01) [7–17]	11.40 (2.39) [7–17]
Domain/overall	89.73 (9.64) [61–116]	81.29 (11.45) [61–101]
Mullen ELC	80.68 (15.47) [49–115]	76.49 (18.66) [49–109]

*RRB* restricted repetitive behavior, *RBS-R* Repetitive Behavior Scale-Revised, *VABS-II* Vineland Adaptive Behavior Scale, *IS* insistence on sameness, *RSM* repetitive sensory motor, *SIB* self-injurious behavior, *CS* coping skills, *PL* play and leisure time, *IPR* interpersonal relationships, *Mullen ELC* Mullen early learning composite

**Table 2:** Hierarchical multiple regression analyses for RRB Subtype (RBS-R) predicting socialization domain scores (VABS-II) at 24 and 36 months

	Model 1	Model 2	Model 3	Model 4
24 Months				
Sex	−1.145	.745	.563	.677
IQ	.095	.085	.125	.130
RSM		−5.081***	−2.463	−2.331
IS			−3.213	−2.900
SIB				−.607
*F*	.838	8.342***	7.246***	5.743***
*R*^2^	.027	.298	.333	.335
Δ*R*^2^		**.271** ***	.035	.002
36 Months				
Sex	4.340	5.515	5.453	7.341
IQ	.336***	.227**	.224**	.216**
RSM		−6.312***	−6.723**	−5.710*
IS			.501	.356
SIB				−2.139
*F*	7.693**	15.191***	11.058***	9.430***
*R*^2^	.325	.595	.596	.619
Δ *R*^2^		**.270** ***	.000	.023

Standardized regression coefficients. 24 months (*N* = 63). 36 months (*N* = 35). Bold face indicates Δ *R*^2^ that survive the Bonferroni adjusted alpha level (*α* = .008)

*RRB* restricted repetitive behavior, *RBS-R* Repetitive Behavior Scale-Revised, *VABS-II* Vineland Adaptive Behavior Scale, *IS* insistence on sameness, *RSM* repetitive sensory motor, *SIB* self-injurious behavior, *IQ* Mullen early learning composite

* *p* < .05.

** *p* < .01.

*** *p* < .001

**Table 3:** Multiple linear regression analyses for RRB subtype (RBS-R) predicting socialization subdomains (VABS-II) at 24 and 36 months

	RRB	IS		RSM		SIB	
Subdomain	Age	24	36	24	36	24	36
CS	βRRB	−.71**	−.68*	−.59*	−1.06***	−.37	−.67*
	BIQ		.04*		.03		.04*
	*R* ^2^	.12	.31	.08	.45	.03	.30
	*F*	7.96**	7.08**	5.36*	13.02***	1.99	6.91**
PL	βRRB	−.90**	−.93*	−1.29***	−1.51***	−.76*	−.90*
	βIQ		.05*		.03		.05*
	*R* ^2^	.13	.29	.27	.45	.09	.27
	*F*	8.88**	6.38**	21.99***	12.96***	6.22*	6.07**
IPR	βRRB	−.90***	−.80*	−.76**	−.77*	−.75**	−.31
	βIQ		.07***		.06**		.07***
	*R* ^2^	.20	.45	.14	.43	.14	.35
	*F*	15.19***	13.02***	10.18**	12.18***	9.86**	8.79***

Standardized regression coefficients. 24 months (*N* = 63). 36 months (*N* = 35). Bold face indicates βRRB that survive the Bonferroni adjusted alpha level (*α* = .003)

*RRB* restricted repetitive behavior, *RBS-R* Repetitive Behavior Scale-Revised, *VABS-II* Vineland Adaptive Behavior Scale, *IS* insistence on sameness, *RSM* repetitive sensory motor, *SIB* self-injurious behavior, *CS* coping skills, *PL* play and leisure time, *IPR* interpersonal relationships, *IQ* Mullen early learning composite

* *p* < .05.

** *p* < .01.

*** *p* < .001
