# Supplementary material for: Relations of Restricted and Repetitive Behaviors to Social Skills in Toddlers with Autism
Source: J Autism Dev Disord. 2021 May 6;52(4):1423–34. doi: 10.1007/s10803-021-05014-8 (PMC8571122; doi:10.1007/s10803-021-05014-8)
Supplement: Supplementary file 1 — Supplementary file1 (DOCX 31 kb) [file 10803_2021_5014_MOESM1_ESM.docx]

**Supplemental Table 1**

*Multiple Linear Regression Analyses for RRB (RBS-R) Predicting Socialization Subdomains (VABS-II) at 24 Months*

| RRB | IS | | | | RSM | | | SIB |
| --- | --- | --- | --- | --- | --- | --- | --- | --- |
|  | Subtype | Comp | Ritual | Same | Subtype | Stereo | Restrict | Subtype |
| CS |  |  |  |  |  |  |  |  |
| $\beta_{RRB}$ | –.71** | –.84** | –.47 | –.66* | –.59* | –.53* | –.56* | –.37 |
| $R^{2}$ | .12 | .16 | .05 | .10 | .08 | .06 | .07 | .03 |
| *F* | 7.96** | 11.87** | 3.27 | 6.86* | 5.36* | 4.23* | 4.71* | 1.99 |
| PL |  |  |  |  |  |  |  |  |
| $\beta_{RRB}$ | –.90** | –.78* | –.78* | –.92** | –1.29*** | –1.29*** | –1.04*** | – .76* |
| $R^{2}$ | .13 | .10 | .10 | .13 | .27 | .26 | .17 | .09 |
| *F* | 8.88** | 6.54* | 6.49* | 9.35** | 21.99*** | 21.7*** | 12.57*** | 6.22* |
| IPR |  |  |  |  |  |  |  |  |
| $\beta_{RRB}$ | –.90*** | –.91*** | –.72** | –.87*** | –.76** | –.66** | –.75** | –.75** |
| $R^{2}$ | .20 | .20 | .13 | .19 | .14 | .11 | .14 | .14 |
| *F* | 15.19*** | 15.60*** | 8.94** | 14.06*** | 10.18** | 7.29** | 9.85** | 9.86** |

*Note*. *N* = 63. Standardized regression coefficients for the 6 RRB subscales (Comp = compulsive, Ritual = ritualistic, Same = sameness, Stereo = stereotyped, Restrict = restricted, SIB = self-injurious behavior) predicting socialization subdomains (CS = coping skills, PL = play and leisure time, IPR = interpersonal relationships) are presented by RRB subtype (IS = insistence on sameness, RSM = repetitive sensory motor, SIB = self-injurious behavior).

RRB = restricted repetitive behavior; RBS-R = Repetitive Behavior Scale-Revised; VABS-II = Vineland Adaptive Behavior Scale.

**p* < .05. ***p* < .01. ****p* < .001.

**Supplemental Table 2**

*Multiple Linear Regression Analyses for RRB (RBS-R) Predicting Socialization Subdomains (VABS-II) at 36 Months*

| RRB | IS | | | | RSM | | | SIB |
| --- | --- | --- | --- | --- | --- | --- | --- | --- |
|  | Subtype | Comp | Ritual | Same | Subtype | Stereo | Restrict | Subtype |
| CS |  |  |  |  |  |  |  |  |
| $\beta_{RRB}$    $\beta_{IQ}$ | –.68*    .04* | –.44     .04* | –.56       .04* | –.70*      .04** | –1.06***     .03 | –1.02**      .03 | –.89**      .04* | –.67*     .04* |
| $R^{2}$ | .31 | .24 | .27 | .32 | .45 | .43 | .38 | .39 |
| *F* | 7.08** | 5.13* | 5.95** | 7.38** | 13.02*** | 11.85*** | 9.93*** | 6.91** |
| PL |  |  |  |  |  |  |  |  |
| $\beta_{RRB}$    $\beta_{IQ}$ | –.93*     .05* | –.86*    .05* | –.36      .06* | –1.09**     .05* | –1.51***     .03 | –1.44***      .03 | –1.28**       .04* | –.90*      .05* |
| $R^{2}$ | .29 | .27 | .19 | .33 | .45 | .42 | .38 | .27 |
| *F* | 6.38** | 5.86** | 3.70* | 7.88** | 12.96*** | 11.48*** | 9.81*** | 6.07** |
| IPR |  |  |  |  |  |  |  |  |
| $\beta_{RRB}$    $\beta_{IQ}$ | –.80**  .07*** | –.46    .07*** | –.93**     .07*** | –.68*     .07*** | –.77*  .06** | –.58    .06** | –.83*    .06*** | –.31    .07*** |
| $R^{2}$ | .45 | .37 | .49 | .42 | .43 | .39 | .45 | .35 |
| *F* | 13.02*** | 9.55*** | 15.15*** | 11.46*** | 12.18*** | 10.21*** | 13.12*** | 8.79*** |

*Note*. *N* = 35. Standardized regression coefficients for the 6 RRB subscales (Comp = compulsive, Ritual = ritualistic, Same = sameness, Stereo = stereotyped, Restrict = restricted, SIB = self-injurious behavior) predicting socialization subdomains (CS = coping skills, PL = play and leisure time, IPR = interpersonal relationships) are presented by RRB subtype (IS = insistence on sameness, RSM = repetitive sensory motor, SIB = self-injurious behavior).

RRB = restricted repetitive behavior; RBS-R = Repetitive Behavior Scale-Revised; VABS-II = Vineland Adaptive Behavior Scale; IQ = Mullen Early Learning Composite.

**p* < .05. ***p* < .01. ****p* < .001.
