# Supplementary material for: Relations of Restricted and Repetitive Behaviors to Social Skills in Toddlers with Autism
Source: J Autism Dev Disord. Author manuscript; Available in PMC 2022 Apr 1. (PMC8571122; doi:10.1007/s10803-021-05014-8)
Supplement: Supplementary file 1 [file NIHMS1704260-supplement-Supplementary_Tables.docx]

**Supplemental Table 1**

*Hierarchical Multiple Regression Analyses for RRB (RBS-R) Predicting Socialization Domain Score (VABS-II) at 24 Months*

| RRB | IS |  |  |  | RSM |  |  | SIB |
| --- | --- | --- | --- | --- | --- | --- | --- | --- |
|  | Subtype | Comp | Ritual | Same | Subtype | Stereo | Restrict | Subtype |
| Step 1    Sex | –1.15 | –1.15 | –1.15 | –1.15 | –1.15 | –1.15 | –1.15 | –1.15 |
| IQ | 1.47 | 1.47 | 1.47 | 1.47 | 1.47 | 1.47 | 1.47 | 1.47 |
| $R^{2}$ | .03 | .03 | .03 | .03 | .03 | .03 | .03 | .03 |
| Step 2    Sex | .15 | –1.10 | .66 | .18 | .74 | 1.06 | –.39 | .53 |
| IQ | 2.33* | 1.91 | 2.30* | 2.37* | 1.32 | 1.29 | 1.42 | 2.37* |
| RRB | –5.24*** | –5.11*** | –4.29*** | –5.15*** | –5.08*** | –4.76*** | –4.49*** | –4.24*** |
| $R^{2}$ | .31 | .31 | .21 | .30 | .30 | .26 | .24 | .21 |
| Δ$R^{2}$ | .29*** | .28*** | .19*** | .27*** | .27*** | .23*** | .22*** | .18*** |
| *F* | 8.93*** | 8.68*** | 5.32** | 8.48*** | 8.34*** | 6.95*** | 6.33*** | 5.16** |

*Note*. *N* = 63. Standardized regression coefficients for the 6 RRB subscales (Comp = compulsive, Ritual = ritualistic, Same = sameness, Stereo = stereotyped, Restrict = restricted, SIB = self-injurious behavior) are presented by RRB subtype (IS = insistence on sameness, RSM = repetitive sensory motor, SIB = self-injurious behavior). 

RRB = restricted repetitive behavior; RBS-R = Repetitive Behavior Scale-Revised; VABS-II = Vineland Adaptive Behavior Scale; IQ = Mullen Early Learning Composite. 

**p* < .05. ***p* < .01. ****p* < .001.

**Supplemental Table 2**

*Hierarchical Multiple Regression Analyses for RRB (RBS-R) Predicting Socialization Domain Score (VABS-II) at 36 Months*

| RRB | IS |  |  |  | RSM |  |  | SIB |
| --- | --- | --- | --- | --- | --- | --- | --- | --- |
|  | Subtype | Comp | Ritual | Same | Subtype | Stereo | Restrict | Subtype |
| Step 1    Sex | 4.34 | 4.34 | 4.34 | 4.34 | 4.34 | 4.34 | 4.34 | 4.34 |
| IQ | 6.28*** | 6.28*** | 6.28*** | 6.28*** | 6.28*** | 6.28*** | 6.28*** | 6.28*** |
| $R^{2}$ | .32 | .32 | .32 | .32 | .32 | .32 | .32 | .32 |
| Step 2    Sex | 5.61 | 6.20 | 4.65 | 5.10 | 5.52 | 6.48 | 4.11 | 8.71* |
| IQ | 5.56*** | 5.74** | 5.73** | 5.79*** | 4.23** | 4.21** | 4.95** | 5.30** |
| RRB | –4.59** | –3.64* | –3.40* | –4.61** | –6.31*** | –5.95*** | –5.48*** | –4.59** |
| $R^{2}$ | .48 | .42 | .41 | .48 | .60 | .56 | .54 | .46 |
| Δ$R^{2}$ | .16** | .09* | .09* | .16** | .27*** | .23*** | .22*** | .13** |
| *F* | 9.54*** | 7.47*** | 7.19*** | 9.79*** | 15.19*** | 13.02*** | 12.16*** | 8.71*** |

*Note*. *N* = 35. Standardized regression coefficients for the 6 RRB subscales (Comp = compulsive, Ritual = ritualistic, Same = sameness, Stereo = stereotyped, Restrict = restricted, SIB = self-injurious behavior) are presented by RRB subtype (IS = insistence on sameness, RSM = repetitive sensory motor, SIB = self-injurious behavior). 

RRB = restricted repetitive behavior; RBS-R = Repetitive Behavior Scale-Revised; VABS-II = Vineland Adaptive Behavior Scale; IQ = Mullen Early Learning Composite. 

**p* < .05. ***p* < .01. ****p* < .001.

**Supplemental Table 3**

*Multiple Linear Regression Analyses for RRB (RBS-R) Predicting Socialization Subdomains (VABS-II) at 24 Months*

| RRB | IS |  |  |  | RSM |  |  | SIB |
| --- | --- | --- | --- | --- | --- | --- | --- | --- |
|  | Subtype | Comp | Ritual | Same | Subtype | Stereo | Restrict | Subtype |
| CS |  |  |  |  |  |  |  |  |
| $β_{RRB}$    $β_{IQ}$ | –.79**    .53* | –.84** | –.56*      .50 | –.75**     .53* | –.59* | –.53* | –.56* | –.47  .49 |
| $β_{Sex}$ |  |  |  |  |  |  |  |  |
| $R^{2}$ | .18 | .16 | .11 | .16 | .11 | .06 | .07 | .09 |
| *F* | 6.49** | 11.87** | 3.61* | 5.87** | 5.36* | 4.23* | 4.71* | 2.83 |
| PL |  |  |  |  |  |  |  |  |
| $β_{RRB}$    $β_{IQ}$ | –.98**    .54 | –.78* | –.88**      .54 | –1.01**     .55 | –1.29*** | –1.29*** | –1.04*** | – .87**  .56 |
| $β_{Sex}$ |  |  |  |  |  |  |  |  |
| $R^{2}$ | .17 | .10 | .14 | .18 | .27 | .26 | .17 | .14 |
| *F* | 6.23** | 6.54* | 4.93* | 6.59** | 21.99*** | 21.7*** | 12.57*** | 4.88* |
| IPR |  |  |  |  |  |  |  |  |
| $β_{RRB}$    $β_{IQ}$ | –.93***    .19 | –.91*** | –.75**      .18 | –.90***     .20 | –.76** | –.66** | –.75** | –.79**  .20 |
| $β_{Sex}$ |  |  |  |  |  |  |  |  |
| $R^{2}$ | .21 | .20 | .14 | .20 | .14 | .11 | .14 | .15 |
| *F* | 7.90*** | 15.60*** | 4.71* | 7.35** | 10.18** | 7.29** | 9.85** | 5.26** |

*Note*. *N* = 63. Standardized regression coefficients for the 6 RRB subscales (Comp = compulsive, Ritual = ritualistic, Same = sameness, Stereo = stereotyped, Restrict = restricted, SIB = self-injurious behavior) predicting socialization subdomains (CS = coping skills, PL = play and leisure time, IPR = interpersonal relationships) are presented by RRB subtype (IS = insistence on sameness, RSM = repetitive sensory motor, SIB = self-injurious behavior). 

RRB = restricted repetitive behavior; RBS-R = Repetitive Behavior Scale-Revised; VABS-II = Vineland Adaptive Behavior Scale; IQ = Mullen Early Learning Composite. 

**p* < .05. ***p* < .01. ****p* < .001.

**Supplemental Table 4**

*Multiple Linear Regression Analyses for RRB (RBS-R) Predicting Socialization Subdomains (VABS-II) at 36 Months*

| RRB | IS |  |  |  | RSM |  |  | SIB |
| --- | --- | --- | --- | --- | --- | --- | --- | --- |
|  | Subtype | Comp | Ritual | Same | Subtype | Stereo | Restrict | Subtype |
| CS |  |  |  |  |  |  |  |  |
| $β_{RRB}$    $β_{IQ}$ | –.68*      .78* | –.44     .82* | –.56      .80* | –.70*     .81** | –1.06***     .54 | –1.02**     .53 | –.89**      .67* | –.91**    .68* |
| $β_{Sex}$ |  |  |  |  |  |  |  | 1.70* |
| $R^{2}$ | .31 | .24 | .27 | .32 | .45 | .43 | .38 | .39 |
| *F* | 7.08** | 5.13* | 5.95** | 7.38** | 13.02*** | 11.85*** | 9.93*** | 6.68** |
| PL |  |  |  |  |  |  |  |  |
| $β_{RRB}$    $β_{IQ}$ | –.93*     .98* | –.86*  1.00* | –.36    1.06* | –1.09**   1.00* | –1.51***     .63 | –1.44***     .63 | –1.28**       .81* | –1.07*     .89* |
| $β_{Sex}$ |  |  |  |  |  |  |  | 1.19 |
| $R^{2}$ | .29 | .27 | .19 | .33 | .45 | .42 | .38 | .30 |
| *F* | 6.38** | 5.86** | 3.70* | 7.88** | 12.96*** | 11.48*** | 9.81*** | 4.41* |
| IPR |  |  |  |  |  |  |  |  |
| $β_{RRB}$    $β_{IQ}$ | –.80**   1.27*** | –.46  1.33*** | –.93**    1.24*** | –.68*   1.32*** | –.77*   1.14** | –.58   1.19** | –.83*    1.19*** | –.57  1.26*** |
| $β_{Sex}$ |  |  |  |  |  |  |  | 1.80 |
| $R^{2}$ | .45 | .37 | .49 | .42 | .43 | .39 | .45 | .43 |
| *F* | 13.02*** | 9.55*** | 15.15*** | 11.46*** | 12.18*** | 10.21*** | 13.12*** | 7.67*** |

*Note*. *N* = 35. Standardized regression coefficients for the 6 RRB subscales (Comp = compulsive, Ritual = ritualistic, Same = sameness, Stereo = stereotyped, Restrict = restricted, SIB = self-injurious behavior) predicting socialization subdomains (CS = coping skills, PL = play and leisure time, IPR = interpersonal relationships) are presented by RRB subtype (IS = insistence on sameness, RSM = repetitive sensory motor, SIB = self-injurious behavior). 

RRB = restricted repetitive behavior; RBS-R = Repetitive Behavior Scale-Revised; VABS-II = Vineland Adaptive Behavior Scale; IQ = Mullen Early Learning Composite. 

**p* < .05. ***p* < .01. ****p* < .001.
